# Prevalence of Neonatal Sepsis and Associated Factors among Neonates in Neonatal Intensive Care Unit at Selected Governmental Hospitals in Shashemene Town, Oromia Regional State, Ethiopia, 2017

**DOI:** 10.1155/2018/7801272

**Published:** 2018-08-02

**Authors:** Aytenew Getabelew, Mihret Aman, Endashaw Fantaye, Tomas Yeheyis

**Affiliations:** ^1^Department of Nursing, College of Medicine and Health Science, Ambo University, Ambo, Ethiopia; ^2^Department of Nursing, College of Medicine and Health Science, Arba Minch University, Arbaminch, Ethiopia

## Abstract

**Background:**

Neonatal sepsis is an important cause of morbidity and mortality among neonates in developing countries accounting for 30-50% of total deaths each year. Childhood mortality is often used as broad indicator of the social development or a specific indicator of health conditions of a country.

**Objectives:**

The objective of this study is to assess the prevalence of neonatal sepsis and associated factors among neonates admitted in neonatal intensive care unit at two hospitals in Shashemene town, Ethiopia.

**Method:**

An institution based cross-sectional study with retrospective document review method was conducted in NICUs of two governmental hospitals in Shashemene town. Sample size was calculated by using single population proportion sample formula and the final sample size was 244. The study subject was selected by using systematic random sampling method, and adopted data collection tool was used. Then the collected data was coded and entered in to SPSS for windows version 20.0 for cleaning, editing, and analysis. Binary and multiple logistic regressions have been used to observe the association between independent variables and dependent variable.

**Result:**

The overall prevalence of neonatal sepsis in this study was 77.9%. From this 65% and 35% of neonates developed early onset neonatal sepsis and late onset neonatal sepsis, respectively. This study found out that age of neonates, birth asphyxia, and use of oxygen via mask were significantly associated with neonatal sepsis*. Conclusion and Recommendation*. The most risk factors of neonatal sepsis were identified as age of neonates, birth asphyxia, and use of oxygen via mask strongly associated with prevalence of NS. Based on this results we recommend the concerned body to focus on the prevention of risk factors rather than treating the disease after it occurs.

## 1. Background

Deaths occurring in the neonatal period each year account for 41% (3.6 million) of all deaths in children under 5 years. The majority of these deaths occur in low income countries and almost 1 million of these deaths are attributable to infectious causes including neonatal sepsis, meningitis, and pneumonia. Furthermore, neonatal mortality for different African countries ranges from 68 per 1000 live births in Liberia to 11 per 1000 live births in South Africa [1].

Neonatal sepsis contributes substantially to neonatal morbidity and mortality and is an ongoing major global public health challenge [2]. According to the World Health Organization (WHO), four million newborn children die each year during the first four weeks of their lives. Of these, 75% die prematurely during the first week of life [3, 4]. The major causes of neonatal deaths globally were estimated to be infections (35%), preterm births (28%), intrapartum related complication (24%), and asphyxia (23%). Sepsis is the commonest cause of neonatal mortality and is probably responsible for 30-50% of the total neonatal deaths each year in developing countries [5].

According to the current united nation estimate, the neonatal death reduced by 48% from the 1990 estimate to 28 per 1000 live births in 2013 while the reduction rate of under-five mortality rate was about 67% [6]. The16 EDHS reported that neonatal mortality rate is 29/1000 live birth, which has a reduction from the 2005 EDHS report of 39/1000 live births and 2011EDHS report of 37/1000 live birth.

The common causes of neonatal mortality in Ethiopia are infection, asphyxia, and preterm birth [7]. Many women do not generally seek formal healthcare during pregnancy, child birth, and puerperal. This has a major impact on care seeking for and survival of the new born. Less than third of women receive antenatal care and 90% are assisted by unskilled attendants: TBAs (26%) relatives (58%) or alone (6%). Almost no one (3.5%) receives postnatal care [8].

Newborn survival is an issue of great concern to the world and especially to developing countries. Care for the neonate often receives little attention in maternal and child health programs. Though various efforts have been made by Ethiopian government to reduce neonatal mortality some studies in Gondar, Black Lion specialized hospital and Bishoftu general hospital, show that it is still high. So it is important to do additional research regarding this title typically on associated factors.

Therefore, this research was conducted to find out the prevalence of neonatal sepsis and associated factors in Shashemene town at two governmental hospitals, South Ethiopia. In addition it will provide opportunity for stake holders to reduce the problem by working on identified factors.

## 2. Methods and Materials

### 2.1. Study Setting, Design, Period

The study is conducted in Shashemene town, Oromia Regional State, South Ethiopia, at two governmental hospitals. Shashemene referral hospital which is found in Shashemene town situated 238 KM from Addis Ababa and 9 KM North from Shashemene town and Melkaoda general hospital which is located in near Shashemene town, 250 km away from the capital city, Addis Ababa. This study was carried out using institution based cross section study with retrospective document review from February 5, 2017 to February 30, 2017.

### 2.2. Sample Size Determination and Sampling Technique

The sample size was determined by using single population proportion formula and the proportion was taken from the previous literature in Ethiopia. According to study conducted at Black Lion specialized hospital, the prevalence of neonatal sepsis was 44.7% [9].

By considering 95% confidence interval (CI) and 5% marginal error the, sample size was calculated as follows:

(1) $$\begin{array}{c} Where\;\;n={\left(\;Z_{\partial /2}\;\right)}^{2}\frac{p\left(1-p\right)}{d^{2}} \end{array}$$

  n= required sample size
  Z= the standard normal deviation at 95% confidence interval; =1.96
  P= proportion of neonatal sepsis among neonates admitted in NICU with prevalence of 44.7
  d= margin of error that can be tolerated, 5% (0.05)
  1-p = proportion of population that do not possess the character of interest.

Therefore,

(2) n=Z∂/22p1−pd2=1.9620.4470.5530.052=380

By considering nonresponse rate 5%, our total sample size was 399 neonates.

Total number of our study population was less than 10,000, which means that the total number of neonates admitted to this two hospital within one year was 626, so correction formula was used; the final sample size was** 244** neonates.

**Figure 1:**
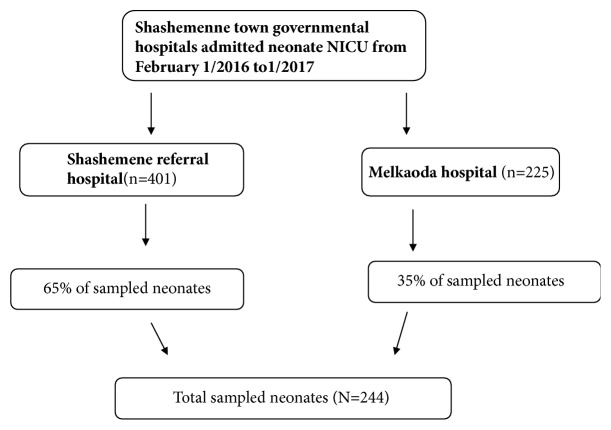
Schematic representation of sampling procedure of the study.

The study populations was neonate's card who were admitted and treated in NICUs of two government hospitals in the area. These two hospitals are selected purposively because these are the only hospitals in the area which have NICU and render service in this arena. The number of study subjects for each hospital was allocated proportionally after identifying the number of admissions in each hospital in the last one year. Study Subjects were selected by using systematic random sampling method (every Kth) after calculating the “Kth” value by dividing the total number of neonates within the last one year to the required sample size which was allocated proportionally for each hospital and the first subject is selected by lottery method. Medical records with full information were used and if the chart is missing the next in the queue was used (Figure 1).

In this study neonatal is asserted when a medical diagnosis of the neonate is stated as ‘neonatal sepsis' by the physician in the neonate's medical record chart.

### 2.3. Instrument and Data Collection Technique

The data was collected using checklist prepared by reviewing different literatures; the check list contains contain three parts: sociodemographic characteristics; maternal information; and part neonatal information for neonatal sepsis. Records of neonates for the last one year (from February 1/ 2016 to February1/2017) were reviewed. This involves going through log book records of neonates with the diagnosis of sepsis between the times from 2016 to 2017. The medical files were traced using the patient card numbers on the log book registry. If there was incomplete maternal information on the neonatal card, the maternal card was traced by using neonatal card number.

### 2.4. Data Analysis

After the data was collected, questionnaires were reviewed and organized by investigators. The data were entered after defining variables and analyzed using SPSS v. 20.0 statistical software. Binary Logistic regression was performed. COR (crude odds ratio) along with 95% confidence interval was used to determine the existence of an association between independent and dependent variables. Then multivariate logistic regression was used to decrease the effect of confounding factors. Statistical significance was declared with p value less than 0.05 for multivariable and 0.25 for bivariate logistic regressions. Finally, the result is presented using tables, texts, and other pictorial representation.

### 2.5. Data Quality Control

Before the data collection, the data collectors made sure that they have a common understanding of the questionnaire and the questionnaire was translated to Amharic and back to English. After data collection, internal consistency was checked by cross-checking the collected data within on every day of data collection.

### 2.6. Ethical Consideration

Ethical clearance was sought from Ambo University, College of Health Science and Department of Nursing Ethical Review Committee. After explaining the purpose and the possible benefit of the study, permission to gather data was obtained from the medical directors of respective hospitals and heads of the neonatal intensive care unit. The respondent's privacy and confidentiality of the information were assured throughout the study procedure.

## 3. Results

### 3.1. Sociodemographic Characteristics

Among 244 reviewed cards of neonates, 181 (74%) of them were aged between 0 and 7 days while 63 (26%) of neonates were aged between 8 and 28 days. Among neonates who enrolled in this study more than half of them were male 142 (58.2%) and the rest are females. Majority (37.7%) of the mothers were aged between 20 and 24 years (Table 1).

### 3.2. Prevalence of Sepsis and Maternal Risk Factors for Neonatal Sepsis

**Figure 2:**
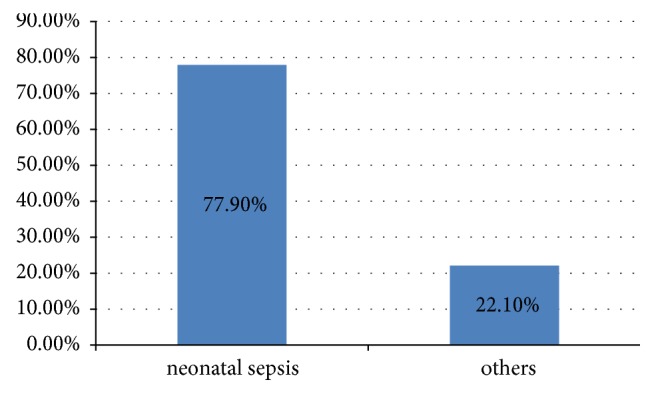
Diagnosis of neonatal sepsis and others cases, in Shashemene referral hospital and Melkaoda General Hospital.

Among 244 neonates who were admitted in NICU 190(77.9%) had neonatal sepsis (Figure 2).

From this were 123 (64.7%) who had early onset neonatal sepsis and 67 (35.3%) who had late onset neonatal sepsis. Among 244 sampled neonates 22 (9%) of their mothers had history of UTI and among this 14 (5.7%) neonates had developed neonatal sepsis. Among a total of sampled neonates 17 (7%) their mothers had history of meconium stained amniotic fluid. Out of this 10 (4%) of them developed neonatal sepsis. Concerning place of delivery 181 (75%) of neonates were delivered in hospital and, out of this, 144 (79.6%) neonates developed neonatal sepsis. About 36 (14.6%) were delivered in health center; from this 27 (11%) of neonates had neonatal sepsis (Table 2).

### 3.3. Neonates Risk Factors for Neonatal Sepsis

Among the total neonates 155 (63.5%) neonates had normal birth weight and 62 (25%) were of low birth weight. Out of normal birth weight and low birth neonates about 52.9% and 17.6% neonates had developed neonatal sepsis, respectively. Among the total 244 neonates 65 (26.6%) neonates were reported to have gestational age less than 37 weeks and 42 (17.2%) of them had developed neonatal sepsis. Referring to APGAR score about 132 (54.1%) neonates had APGAR score>7 and out of them 114 (59%) of them developed sepsis. About 112 (45.9%) neonates had APGAR score less than 7 and 76 (31.1%) of them were presented with neonatal sepsis (Table 3).

### 3.4. Medical Risk Factors of Neonatal Sepsis

From the total 244 study units 98 (40.2%) neonates were reported to have mechanical ventilation; out of this 70 (28.7%) of them had neonatal sepsis. Among the total study population more than three-fourth of them 143 (58.6%) had oxygen via catheter and 106 (74.1%) of these neonates developed neonatal sepsis (Table 4).

### 3.5. Factors Associated with Neonatal Sepsis

On this study, bivariate logistic regression analysis revealed that age of neonates, maternal history of meconium aspiration, birth asphyxia, use of oxygen via mask, low birth weight, and gestational age less than 37 weeks show significant association with neonatal sepsis. Multivariate logistic regression neonates whose age were less than seven days were 3 times more likely to develop neonatal sepsis compared with the age of neonates greater than eight days of age (AOR = 3.01 with 95% CI (1.148,7.89). Neonates who had birth asphyxia were 3.54 times more likely to have neonatal sepsis compared to those who did not have birth asphyxia (AOR=3.54 95%CI (1.57,7.984)). Finally the neonates who used oxygen via mask were 2.859 times highly at risk to develop neonatal sepsis compared to neonates those who did not used it at birth [AOR=2.859 with 95%CI (1.300,6.289)] (Table 5).

## 4. Discussion

In this study the overall prevalence of neonatal sepsis was 77.9%. The study conducted in Iran shows that prevalence of neonatal sepsis was 51.8% [10]. This difference could be due to smaller sample size and difference in sociodemographic status of population as well as accessibility of health facility and differences in the definition of neonatal sepsis among the studies. Although other studies in Egypt reported that prevalence of neonatal sepsis was 40.7% [11], this difference could be due to the fact that they use confirmed laboratory results based on blood culture. Although this study finding is greater than the study conducted in Black Lion specialized hospital in 2010 which was 44.7% [12], this difference might be because culture is not used for diagnosing neonatal sepsis in this study and clinical features alone were used in both hospitals. In addition, the other possible reason might be that the area and the community are far from the center of the country and from health facilities which in turn decreases of the population and increases risk of harmful traditional practices on children. There was another study conducted in Douala Cameroon in urban district hospital in 2014 which reported that the prevalence of NS was 79.1% [13].

## 5. Conclusion

In the study we found that among a total of neonates admitted in Shashemene hospital, the overall prevalence of neonatal sepsis by physicians diagnosis from the clients medical record was 77.9% within the last one year from two hospitals. The factors associated with neonatal sepsis were birth asphyxia, age being less than 7 days, and use of oxygen via mask.

## Figures and Tables

**Table 1:** Sociodemographic characteristics of neonates and mothers unit at selected governmental hospital in Shashemene town, Oromia regional state, south ethiopia, 2017G.

Address		Frequency	sepsis		
			Early(%)	Late onset	Others
Shashemenne		121(49.6%)	58(23.7%)	33(13.5%)	30(12.2%)

Others		123(50.4%)	65(26.6%)	34(13.9%)	24(9.8%)

Age of neonates	0-7 day	181(74%)	134(55%)	-	47(19%)
	8-28 days	63(26%)	-	56(23%)	7(3%)

Sex of neonate	Male	142(58.1%)	75(30.7%)	35(14.3%)	32(13.1%)
	Female	102(41.8%)	48(19.6%)	32(13.1%)	22(9.1%)

Maternal age	<19	42(17.2%)	21(8.6%)	12(4.9%)	9(3.7%)
	20-24	92(37.7%)	51(20.9%)	26(10.6%)	15(6.2%)
	25-29	75(30.7)	39(15.9%)	17(6.9%)	19(7.7%)
	30-35	31(12.7%)	10(4.1%)	10(4.1%)	11(4.5%)
	>35	4(1.6%)	2(0.8%)	2(0.8%)	0(0%)

**Table 2:** Distribution maternal risk factors for neonatal sepsis among neonates admitted in Shashemenne referral hospital and Melka Oda district hospital in NICU wards, Ethiopia, 2017.

VARIABLE		Frequency	Sepsis		
			EONS	LONS	OTHERS
History of maternal UTI	Yes	22(9%)	7(2.9%)	7(2.9%)	8(3.4%)
	No	222(91%)	116(47.5%)	60(24.6%)%)	46(18.9%)

History of antenatal care	Yes	192(78.7%)	101(41.4%)	52(21.3%)	39(16%)
	No	52(21.3%)	22(9%)	15(6%)	15(6%)

History of Maternal Fever	Yes	36(14.8%)	14(5.7%)	11(4.5%)	11(4.5%)
	No	208(85.2)	109(44.7%)	56(23%)	43(17.6%)

History of foul smelling liquor	Yes	10(4.1%)	5(2%)	2(0.8%)	3(1.2%)
	No	234(95.9%)	118(48.4%)	65(26.6%)	51(20.9%)

History of Chorioamnionitis	Yes	12(4.9%)	6(2.5%)	3(1.2%)	3(1.2%)
	No	232((95.1%)	117(48%)	64(26%)	51(20.9%)

Meconium stained amniotic fluid	Yes	17(7%)	7(2.9%)	3(1.2%)	7(2.9%)
	No	227(93%)	116(47.5%)	64(26%)	47(19%)

History of premature rupture of membrane	Yes	52(21.3%)	18(7%)	9(3.7%)	9(3.7%)
	No	192(78.7%)	100(41%)	49(20%)	43(17.6%)

Duration of PROM	<18 hrs	174(71.3%)	96(39%)	42(17.2%)	36(14.8%)
	>18 hrs	24(9.8%)	10(4%)	8(3.2%)	6(2.5%)

Parity	Primi	99(40.6%)	52(21.3%)	23(9.4%)	24(%)
	Multipara	145(59.4%)	71(9.8%)	44(18%)	30(12.3%)

Mode of delivery	S/V	181(74.2	86(35.2%)	53(21.7%)	42(17.2%)
	C/S	52(21.3%)	32(13%)	12(5%)	8(3.3%)
	Vacuum(I)	11(4.5%)	5(2%)	2(0.8%)	4(1.6%)

Place of delivery	Hospital	181(75%)	98(40%)	46(18.9%)	39(16%)
	Health Center	36(14.6%)	18(7.3%)	9(3.7%)	9(3.7%)
	Clinic	6(2.5%)	3(1.2%)	2(0.8%)	1(0.4%)
	Home	19(7.8%)	4(1.6%)	14(5.7%)	1(0.4%)

Duration of labor	<6 hrs	31(12.7%)	10(4%)	13(5.3%)	8(3.3%)
	**6-12 hrs**	**74(32.6**%**)**	39(16%)	19(7.8%)	16(7%)
	12-24 hrs	73(32.1)	41(6.6%)	18(7.4%)	14(5.7%)
	>24 hrs	16(7%)	12(5%)	0(0%)	4(1.6%)

Diagnosis on admission			123(50.4%)	67(27.5%)	52(22.1%)

**Table 3:** Birth related factors predisposing a neonate for neonatal sepsis among neonates admitted in Shashemenne referral hospital and Melka Oda district hospital in NICU wards, Ethiopia, 2017.

		Sepsis			
Variable		Frequency	EONS	LONS	OTHERS
Birth weight	LBW<2.5 kg	69(28.3%)	25(10.2%)	21(8.6%)	23(9.4%)
	NBW 2.5 kg	166(68%)	93(38.1%)	44(18%)	29(11.9%)
	Over,>4 kg	9(3.7%)	5(2%)	2(.8%)	2(0.8%)

Gestational age	Pre-term <37wks	65(26.6%)	21(8.6%)	21(8.6%)	23(9.4%)
	Term37-42wks	179(73.4%)	102((41.8)	46(18.9%)	31(12.7%)

Birth asphyxia	Yes	56(23%)	21(8.6%)	10(4%)	25(10.2%)
	No	188(77%)	102(41.8%)	57(23.4%)	29(11.9%)

APGAR score	>7	132(54.1%)	68(27.9%)	46(18.9%)	18(7.4%)
	<7	112(45.9%	55(22.5%)	21(8.6%)	36(14.8%)

Outcome	Died	46(18.9%)	30(12.3%)	10(4%)	6(2.3%)
	Cured	198(81.1%)	123(50.4%)	40(16.4%)	35(14.3%)

**Table 4:** Medical risk factors for neonatal sepsis among neonates admitted in Shashemenne referral hospital and Melka Oda district hospital in NICU wards, Ethiopia, 2017.

Variable		Frequency	Sepsis		
			EONS	LONS	OTHER
Mechanical ventilation	Yes	98(40.2%)	57(23.4%)	13(5%)	28(11.4%)
	No	146(59.8%)	66(27%)	54(22%)	26(10.7%)

Oxygen via nasal catheter	Yes	143(58.6%)	66(27%)	40(16.4%)	37(15%)
	No	101(41.4%)	57(23.4%)	27(11%)	17(7%)

Oxygen via mask	Yes	47(19%)	16(6.6%)	10(4%)	21(8.6%)
	No	197(81%)	107(43.9%)	57(23.4%)	33(13.5%)

Suspected neonatal sepsis And other cases		190(77.9%)			
		54(22.1%)			

NB: P<0.05, CI-95% and *∗* had association.

**Table 5:** Bivariate and multivariate analysis showing the association between neonatal sepsis and others different variables in neonates admitted to Shashemene governmental hospital, NICU, Ethiopia, 2017, GC.

Variable		Neonatal sepsis				
		Yes	No	P value	COR with 95%CI	AOR with 95% CI
Age of neonates	0-7 days	113(46.3%)	68(27.8%)	.025*∗*	2,806(1.196-6.585)*∗*	3.01(1.148-7.89)
	8-28 days	46(18.8%)	17(7%)		1	1

MSAF	Yes	10(4%)	180(73.8%)	.177	2.979(1.054-8.416)	2.223(.697-7.087)
	No	7(29%)	47(19.3%)		1	1

BW	LBW	46(18.9%)	23(9.4%)	.453	1.541(1.233-4.376)	2.302(.261-20.329)
	NW	144(59%)	31(12.7%)		1	1

GA	Preterm	46(18.9%)	23(9.4%)	.277	2.678(1.411-5.083)	2.167(.537-8.745)
	Term	148(60.7%)	31(12.7%)		1	1

BA	Yes	31(12.7%)	25(10.2%)	.000*∗*	4.422(2.288-8.546)	3.540(1.570-7.984)
	No	159(65.2%)	29(11.9%)		1	1

MV	Yes	70(28.7%)	28(11.5%)	.288	1.873(1.017-3.448)	1.466(.724-2.969)
	No	120(49.2%)	26(10.7%)		1	1

OVM	Yes	25(10.2%)	21(8.6%)	.009*∗*	4.348(2.171-8.712)	2.859(1.300-6.289)
	No	164(67.2%)	33(13.5%)		1	1

## Data Availability

The datasets used and/or analyzed during the current study are available for ethical reasons from the corresponding author and coauthors.

## Abbreviations

ART: Antiretroviral treatment
ANC: Antenatal care
AOR: Adjusted Odds Ratio
APGAR: Activity, pulse, Grimace, appearance, and respiration
BA: Birth asphyxia
CI: Confidence interval
COR: Crude Odds Ratio
EDHS: Ethiopia Demographic and Health Survey
EONS: Early onset neonatal sepsis
LBW: Low birth weight
LONS: Late onset neonatal sepsis
MCH: Maternal to child health
MDG: Millennium development goal
MSAF: Meconium stained amniotic fluid
NICU: Neonatal intensive care unit
NMR: Neonatal mortality rate
OPD: Outpatient department
OVM: Oxygen via mask
PROM: Prolonged rupture of membrane
SPSS: Statistical Package for Social Science
TB: Tuberculosis
TTBA: Traditional trained birth attendant
UTI: Urinary tract infection
WHO: World Health Organization.
